# Urinary Metabolomic Profiling Reveals the Effect of Shenfu Decoction on Chronic Heart Failure in Rats

**DOI:** 10.3390/molecules200711915

**Published:** 2015-06-30

**Authors:** Dawei Yang, Xiaoxing Wang, Yaping Wu, Bo Lu, Aifeng Yuan, Carlos Leon, Na Guo

**Affiliations:** 1 Zhong Yuan Academy of Biological Medicine, Liaocheng People’s Hospital/Affiliated Liaocheng Hospital, Taishan Medical University, 67 Dong Chang Xi Lu, Liaocheng 252000, China; E-Mails: daweiyang2011@gmail.com (D.Y.); u_m_c_u_ywu@hotmail.com (Y.W.); 2 Department of Pharmacy, China-Japan Friendship Hospital, Beijing 100029, China; E-Mail: xxshining@163.com; 3 Experimental Research Center, China Academy of Chinese Medical Sciences, Beijing 100700, China; E-Mails: goldlubo@163.com (B.L.); yuanaifeng07@sina.com (A.Y.); 4 Biomedical Engineering School, Carlos III University, Avda Universidad 30, Leganes, Madrid 28911, Spain; 5 Metabolomics, Genome Center, UC Davis, 451 Health Sciences Drive, Davis, CA 95616, USA

**Keywords:** chronic heart failure, Shenfu decoction, metabolomics, GC/TOF–MS, metabolomic pathway analysis

## Abstract

Shenfu decoction (SFD) can be used to treat patients with sign of Yangqi decline or Yang exhaustion related to chronic heart failure (CHF). We conducted a gas chromatography with time-of-flight mass spectrometer (GC/TOF–MS)-based metabolomic study to increase the understanding of CHF and assess the efficacies and mechanisms of SFD in treating CHF induced by coronary artery ligation in rats. Based on unsupervised principal component analysis, there was a clear separation between the CHF and sham surgery group, which revealed that CHF disturbed the metabolism of endogenous substances and significantly altered the urine metabolite fingerprints. After SFD treatment, the metabolomics profile found in CHF was significantly reversed, shifting much closer to normal controls and sham surgery group, indicating that SFD has therapeutic effects in CHF, which is in accordance with the hemodynamic assay results. Metabolomic pathway analysis demonstrated that several pathways including fatty acid biosynthesis, fatty acid elongation, steroid biosynthesis, galactose metabolism, and amino acid metabolism were significantly altered in CHF rats. Therefore, we may infer that SFD shows therapeutic efficacy in CHF by restoring these disturbed metabolic pathways, especially those related to energy metabolism. This study offers new methodologies for increasing the understanding of CHF and systematically characterizing the efficacies and mechanisms of SFD in treating CHF.

## 1. Introduction

Chronic heart failure (CHF) is a common, complex clinical syndrome that arises from structural or functional cardiac disorder, including changes in electrophysiology, contraction, and energy metabolism [1]. Heart failure (HF) is becoming an increasingly common diagnosis with an incidence approaching 10 per 1000 of the population over 65 years of age in developed countries [2]. Even in China, it was reported that the prevalence of HF in the adult population from ten provinces was 0.9% [3]. Because the prognosis for CHF is poor and there are few therapeutic options, HF is even worse than many types of cancer [4]. Meanwhile, there has been an increased hospitalization burden, which drains healthcare expenditures and makes HF a global public health problem.

The most effective and commonly used drugs for the treatment of HF are angiotensin-converting enzyme (ACE) inhibitors, β-adrenoceptor blockers, and digitalis [5,6,7]. The American Heart Association (AHA) and European Society of Cardiology (ESC) have issued and updated the guidelines for the diagnosis and management of CHF [2]. However, HF is still a leading cause of death worldwide [8], therefore it is necessary to seek novel effective drugs for HF. Traditional Chinese Medicine (TCM) has gained popularity for the treatment of complex multifactor diseases because accomplishes an overall therapeutic effect by targeting multiple pathways to improve therapeutic efficacy and reduce drug-related side effects and drug resistance. TCMs such as Shengmai [9], Sini decoction [10], Shuanglong formula [11], and Huangqi injection [12] have potential therapeutic effects in the treatment of cardiovascular diseases. Shenfu decoction (SFD) is an important TCM prescription with a 3:2 ratio of Radix Ginseng and Fuzi (Radix Aconiti Lateralis Preparata). SFD has been used for the treatment of various diseases with signs of Yangqi decline or Yang exhaustion and especially for cardiovascular diseases [13]. The active components of SFD are ginsenoside and aconitine. However, evaluating SFD’s efficacy and mechanism of pharmacological action is difficult because the active compounds are not well characterized and their possible synergistic actions are unknown. Therefore, new approaches are required for the evaluation of efficacy and mechanism(s) of SFD in treating CHF, which can contribute to the rationalization and modernization of TCM use.

Metabolomics is the analysis of a whole array of metabolites in a biosystem under a given set of conditions [14]. Metabolomics uses a top-down strategy to holistically understand metabolic changes of a complete system caused by interventions. This methodology corresponds with the overall therapeutic effect and systemic features of TCMs, and has the potential to improve our understanding of Chinese medicine theory [15,16]. Therefore, metabolomics could provide a new methodology for systematically characterizing the efficacies and mechanisms of SFD in treating CHF.

Recent advances in mass spectrometry (MS) [17] and nuclear magnetic resonance (NMR) [18] spectroscopy have helped further develop metabolomics. Although NMR is one of the most commonly used technologies in metabolomics research, the high selectivity with low-detection limits of mass spectrometry makes it an ideal tool for metabolomic applications. Especially, the GC/TOF–MS-based platform is a powerful technique in metabolomics because of its peak resolution, reproducibility, and availability of mass spectral libraries for product identification [19,20,21].

The goal of this study was to investigate the biochemical changes in CHF and therapeutic effects and mechanisms of SFD by means of GC–MS-based metabolomics. Urine samples were collected from the established CHF rat model, controls, sham surgery group and SFD-treated group and subjected to metabolomics analysis. Multivariate statistical analysis was used to interpret metabolic changes and to better understand CHF and characterize the efficacies and mechanisms of SFD in treating CHF.

## 2. Results and Discussion

### 2.1. Hemodynamic Assessment

As shown in Table 1, eight weeks after acute MI induced by coronary artery ligation, the rats showed significant decreases in left ventricular systolic pressure (LVSP), left ventricular end diastolic pressure (LVEDP), and ± dp/dtmax compared with the sham and controls, which indicated successful CHF model establishment. However, after pharmacologic interventions with SFD for 3 weeks, a significant improvement in LVSP, LVEDP, and ± dp/dtmax was observed compared with CHF rats, without significant differences compared to sham and control groups. Therefore, we conclude that SFD can exert therapeutic effects on CHF.

**Table 1:** Summary of hemodynamic data ($\bar{\text{X}}$± s, *n* = 6)

Time (week)	Group	LVSP (mmHg)	LVEDP (mmHg)	+dp/dtmax (mmHg)	−dp/dtmax (mmHg)
8	Normal Controls	174.70 ± 2.06	−16.68 ± 2.42	7455.01 ± 643.01	−4964.79 ± 749.17
	Sham Surgery	152.54 ± 27.96	−18.61 ± 4.85	5916.10 ± 779.03	−4677.67 ± 656.71
	CHF group	120.81 ± 11.40 ^Δ,^*	2.09 ± 0.10 ^Δ,^*	2622.39 ± 744.60 ^Δ,^*	−2357.32 ± 482.95 ^Δ,^*
11	Normal Controls	137.33 ± 13.27	−5.38 ± 3.01	12019.08 ± 732.35	−9542.12 ± 1101.26
	Sham Surgery	128.20 ± 6.98	−7.73 ± 3.43	9312.59 ± 1312.69	−8106.91 ± 634.04
	CHF group	105.46 ± 4.37 ^Δ,^*	2.84 ± 0.74 ^Δ,^*	6694.55 ± 712.63 ^Δ,^*	−6925.12 ± 483.54 ^Δ,^*
	SFD-treated	125.28 ± 6.25 ^#^	−2.60 ± 1.64 ^#^	8453.47 ± 979.84 ^#^	−8565.22 ± 942.11 ^#^

^Δ^ represents *P* < 0.05 between the CHF and sham surgery groups; * represents *P* < 0.05 between the CHF and normal control groups; ^#^ represents *P* < 0.05 between the SFD-treated and CHF groups.

### 2.2. Urinary Metabolite Profiling in Rats by GC–MS

**Figure 1:**
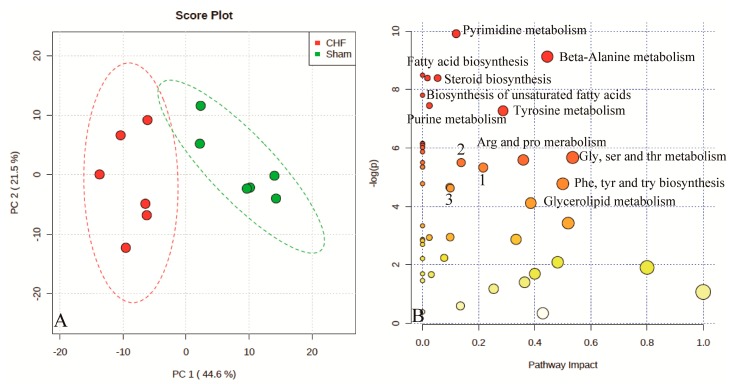
Score plots for principle component analysis model of rat urine data in the CHF and Sham surgery groups (A) Red dots, CHF group; Green dots, Sham surgery group; Metabolomic pathway analysis overview (B) indicating selected metabolic pathways that are significantly affected between CHF and sham surgery group. Size of the node indicates the pathway impact (based on the impact of each identified metabolite in a given pathway). The node color is graded depending on its *p*-value from pathway enrichment analysis. 1, aminoacyl-tRNA biosynthesis; 2, starch and sucrose metabolism; 3, primary bile acid biosynthesis.

Urinary metabolomic profiling from different rat groups were analyzed using GC–MS, including CHF, sham, controls, and SFD group, to better understand the intracellular metabolic changes. A total of 254 metabolites were detected, 143 of which were identified by matching the mass spectra and retention indices in Fiehnlib [22] (Supplementary Materials S1). An unsupervised PCA can discriminate between the CHF and sham groups, which demonstrates that the CHF rat model has different metabolic profiling compared to the sham group (Figure 1A). The differences in the metabolite profile explained by the first principal component were 44.6% and the second principal component was 21.5%.

To interpret the metabolic data from these two groups in a biologically meaningful manner, enrichment and metabolite topology analysis using metabolomic pathway analysis was performed on identified metabolites. An overview of pathway analysis is shown in Figure 1B. The pathway analysis contains all the matched pathways arranged by *p*-value on the Y-axis, based on pathway enrichment analysis, and pathway impact values on the X-axis, from pathway topology analysis. Meanwhile, the node color is graded depending on the *p*-value and the node size is determined based on pathway impact values. Therefore, many metabolic pathways including pyrimidine metabolism, steroid biosynthesis, fatty acid biosynthesis, and amino acid metabolism are significantly affected. These findings demonstrate that more than 20 metabolic pathways were significantly dysregulated in CHF rats compared with the sham group (Supplementary Materials S2). To investigate the therapeutic effects and mechanisms of SF decoction, further investigation is required.

### 2.3. Evaluation of SFD Therapeutic Efficacy

**Figure 2:**
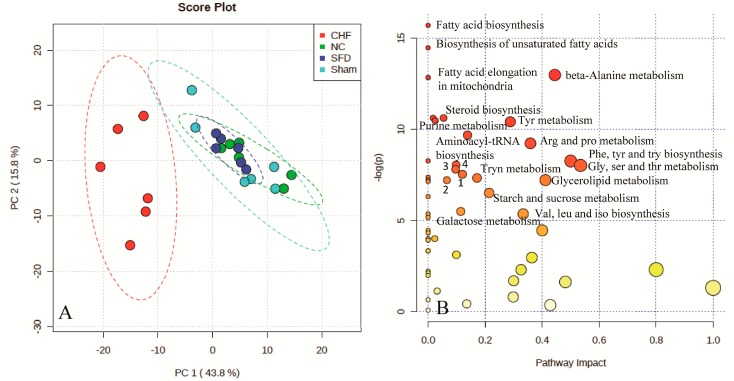
Scores plots for principle component analysis model of rat urine data in the CHF, sham surgery, normal control, and SFD-treated groups (A); Metabolomic pathway analysis overview (B) indicating select metabolic pathways that are significantly affected among the four groups: 1, pyrimidine metabolism; 2, glycerophospholipid metabolism; 3, primary bile acid biosynthesis; 4, glutathione metabolism.

To investigate the global metabolism alterations and evaluate the therapeutic effects of SFD in rats, PCA was used to distinguish metabolic differences of the normal control, CHF model, sham surgery, and SFD groups at week 11 (Figure 2A). In this study, there was a clear separation between CHF and the three other groups including controls, sham surgery, and the SFD-treated group. The metabolic profiling of the control and sham surgery groups had similar projections, which indicates a similar status of the two groups. After SFD treatment, the metabolic profile that was observed in the CHF group was significantly altered from its previous state, and overlapped with the normal control and sham surgery groups. This suggested that SFD had a significant effect on CHF rats, which is also apparent in the hemodynamic assessment.

**Figure 3:**
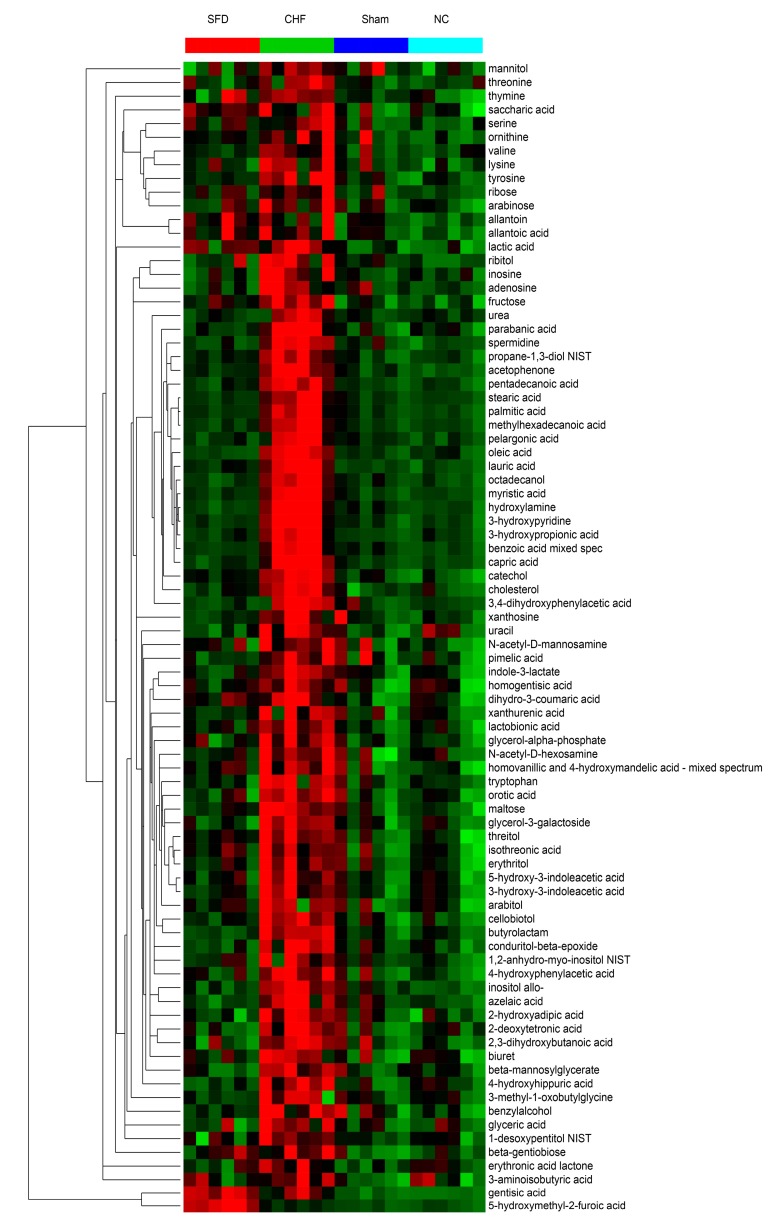
Heat maps showing the 84 significantly-altered metabolites based on ANOVA. Color from green to red represent the intensity of these metabolites from low to high levels in the analysis. CHF, CHF group; NC, normal control group; Sham, sham surgery group; SFD, SFD-treated group. Red dots, SFD-treated group (SFD); Green dots, CHF group (CHF); Light blue dots, normal control group (NC); Dark blue dots, sham surgery group (SS).

Cluster analysis and heat map showed all metabolites that were significantly altered in the dataset (Figure 3). In this depiction, changes in the metabolites in the individual replicates for each group are evident and 84 metabolites were selected based on ANOVA (Supplementary Materials S3). Metabolomic pathway analysis was used to find significantly disturbed metabolic pathways among the four groups (Figure 2B). Fatty acid biosynthesis, fatty acid elongation, steroid biosynthesis, aminoacyl-tRNA biosynthesis, glycerolipid metabolism, galactose metabolism, and amino acid metabolism were significantly altered among the four groups. The complete disturbed metabolic pathways can be found in Supplementary Materials S4. Because CHF has a different metabolic profile than that in the sham group, and the SFD-treated group is overlapped with the sham and control groups, we may infer that SFD has therapeutic efficacy for CHF by restoring some altered metabolic pathways.

### 2.4. Discussion

In the present study, we investigated alterations in urinary metabolomics using GC-MS in rats with CHF induced by coronary artery ligation. In addition, we demonstrated the protective effects of SFD against CHF in rats, which caused significant restoration of urinary metabolic profiles. This alteration establishes a foundation for further investigation into the key mechanisms of SFD in cardiovascular protection.

From GC–MS, we found significant dysfunctions in multiple pathways in CHF rats. As shown in Figure 3, 84 urinary metabolites were disregulated after CHF, including fatty acids, amino acids, saccharides and polyols, and other endogenous small molecules, which are distributed in more than 20 metabolic pathways (Figure 1A and Figure 2A).

**Figure 4:**
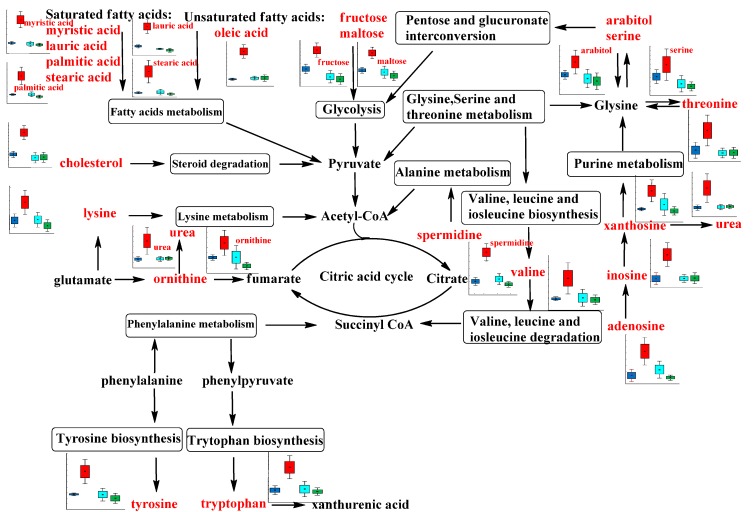
Network of the disregulated metabolites from energy metabolism related pathways from urine samples changing for the CHF, normal control, sham surgery, and SFD-treated groups. Metabolite names in red indicate that they are detected in our study. Box-and-whisker plots of biomarkers were based on ANOVA analysis. Gray color, normal control group; Blue color, sham surgery group; Red color, CHF group; Green color, SFD-treated group.

Among these alterations, the most obvious abnormalities occurred in energy metabolism. This could be because advanced HF is associated with global suppression of metabolic fuel intake and impaired myocardial energy expenditure [23]. We detected elevated levels of seven amino acids (lysine, valine, tyrosine, tryptophan, serine, threonine and ornithine), 11 fatty acids (pentadecanoic acid, capric acid, octadecanol, oleic acid, myristic acid, lauric acid, palmitic acid, stearic acid, pelargonic acid, cholesterol, and methylhexadecanoic acid), and seven saccharides (allo-inositol, fructose, maltose, lactobionic acid, ribitol, arabitol, and glycerol-3-galactoside) in urine of CHF rats compared with the sham group (Figure 4).

All these detected compounds are intermediates in energy metabolism (Figure 4). For example, valine is a branched-chain amino acid, which is an important substrate for biosynthesis of ketone bodies, and glucose, in addition to its role as building block for peptide synthesis [24]. Besides the disturbance of energy metabolism after CHF, these molecules may be useful biomarkers to evaluate pathological status and explore pathophysiological mechanisms. Recently, it was reported that plasma lactate and cholesterol were the major discriminating metabolites predicting mortality in patients with acute HF and lower total cholesterol levels have been associated with increased mortality of patients with various diseases [25]. In our studies, we have found elevated cholesterol and lactate level in the urine of CHF rats, which suggests accelerated excretion of cholesterol. The saturated fatty acids, especially palmitic acid and lauric acid, could activate antigen-presenting cells, and induce the production of inflammatory mediators in coronary artery endothelial and smooth muscle cells, which contribute to vascular inflammation [26,27]. The significantly higher levels of excretion of these saturated fatty acids in CHF rats indicates that abnormal fatty acid biosynthesis and elongation may play an important role in the formation of advanced HF.

The endogenous small molecules inosine, adenosine, xanthosine, and urea, which were significantly higher in CHF rats compared with sham group rats, are all involved in purine metabolism. The intracellular concentrations of these molecules increase when there is a mismatch between ATP synthesis and degradation, as in ischemia or hypoxia [28]. Therefore, these findings reconfirmed the impaired energy metabolism of CHF rats. In addition, adenosine could act as an extracellular signaling molecule with beneficial effects in the coronary microcirculation, such as inducing vasodilation [29]. Several studies have demonstrated mixed findings of adenosine in protecting the myocardium from ischemia via its potent vasodilatory effects and possibly by anti-inflammatory and antiplatelet properties [30]. Therefore, an additional study on the mechanism of the higher level of urinary adenosine in CHF rats should be carried out.

Erythritol is a natural C4 polyol that is not metabolized for energy and excreted unchanged in the urine of humans. Boesten *et al.* showed that erythritol is an excellent hydroxyl radical scavenger and protects endothelial cells from hyperglycemic conditions via effects on multiple targets *in vitro* [31]. In rats with CHF induced by coronary artery ligation, we also found significant differences in the excretion of erythritol between model animals and the sham group. This difference suggested disturbed metabolism of the polyol erythritol when exposed to ischemic or hypoxic conditions, and may contribute to the pathogenesis of CHF.

Besides the discovery of novel biomarkers to increase the understanding of CHF, this metabolomic study based on GC–MS can also clarify some aspects of the therapeutic effects and mechanisms of SFD. In addition to the improvement of cardiac function in CHF rats, the disordered metabolite profiles in urine after CHF were all significantly reversed after SFD treatment. These changes imply that SFD may functionally intervene in several of the pathways of energy metabolism. Although they have been used for thousands of years, few TCMs are well characterized. Many do not demonstrate equal efficacy as that of Western drugs because of the complexity of their active components, unclear synergistic/antagonistic effects, and lack of effective and standard study methods [32]. However, metabolomics, one of the systems biology approaches, provides new insights into exploring the pharmacological mechanisms of TCM. In addition, a growing body of evidence suggests that metabolic remodeling might play a crucial role in the pathophysiology of CHF. Therefore, improvement in energy metabolism is likely to be the most significant pharmacological mechanism for the treatment of CHF with SFD [33].

## 3. Experimental Section

### 3.1. Ethics Statement

This study approach was approved by the Institutional Committee for the Ethics of Animal Care and Treatment in Biomedical Research (Beijing, China). All animal experiments were performed in accordance with the Guide for the Care and Use of Laboratory Animals published by the US National Institutes of Health (NIH publication No. 85-23, revised 1996).

### 3.2. Chemical Reagents and Materials

Acetonitrile and isopropanol for the extraction were purchased from J.T. Baker (Center Valley, PA, USA). Pyridine and *N*-methyl-*N*-(trimethylsilyl)trifluoroacetamide (MSTFA) were purchased from Pierce (Rockford, IL, USA). Chloroform, methanol, and methoxyamine hydrochloride (Sigma-Aldrich, St. Louis, MO, USA) were used for derivatization. Fatty acid methyl esters (FAME) markers were purchased from Sigma-Aldrich.

Ginseng (Panax ginseng CA Mayer) was purchased from Liaoning Luyuan Pharmaceutical Co., Ltd (Dalian, China). Processed aconite root (Radix Aconiti lateralis praeparata) was purchased from Tong-Ren-Tang Pharmaceutical store (Beijing, China). Panax ginseng and the prepared aconite root were authenticated by Xirong He, Institute of Traditional Chinese Medicine, China Academy of Chinese Medical Sciences. Shen-Fu Decoction (SFD) was prepared by combining Panax ginseng and the processed aconite root (at a ratio of 3:2). Dried and pulverized white ginseng (1800 g) and the processed aconite root (1200 g) were ground and then refluxed three times with 30 L of water for 60 min at 100 °C. After cooling and filtering, the solutions were condensed under vacuum and freeze-dried. To minimize the variability of active ingredients in the decoction and ensure repeatable and reproducible therapeutic effects, the decoction was carefully quality controlled. Ginsenoside Re, Rg1, Rb1, Rc, Rb2, Rd, Rf, aconitine, hypaconitine, and mesaconitine in SFD were quantified for quality control, as previously described [34].

### 3.3. Animal Models

Sixty-five male Sprague-Dawley rats (body weight, 200 ± 20 g) provided by Institute of Laboratory Animal Science, Chinese Academy of Medical Sciences, were kept in an animal room with temperature and relative humidity of 22 ± 3 °C and 55% ± 5%, respectively. A light cycle of 12 h on and 12 h off was set. All animals were allowed to acclimatize in metabolism cages for 5 days before treatment. First, we used the coronary artery ligation model to induce myocardial infarction (MI) [35,36]. Because of its clinical relevance and relative simplicity, MI in the rat is a widely used small animal model of HF [37]. Twelve rats were included in the control group. Six rats survived throughout the experiment and six rats were sacrificed for hemodynamic experiments in the eighth week (final normal control group, *n* = 6). Then, 53 rats were randomly selected to undergo surgery or sham surgery. The rats were anesthetized via the intraperitoneal injection of 3% pentobarbital sodium (50 mg/kg).

Following thoracotomy in the fourth and the fifth intercostal space, the heart was exposed. A silk suture was placed under the left main coronary artery about 2 mm from its origin, and the vessel was ligated. Negative pressure was then applied, and the chest wall was sutured closed. Rats were considered to have developed CHF 8 weeks after coronary artery ligation by hemodynamic evaluation (Table 1). Twelve rats in the sham group were treated in a similar manner except that the coronary artery was not ligated, and six rats were sacrificed for hemodynamic experiments in the eighth week (final sham surgery group, *n* = 6). Of the 41 CHF rats, twenty-three rats died after surgery and six rats were sacrificed for hemodynamic experiments in the eighth week. Therefore, 12 rats were left for further urine sample collection (CHF group and SFD treated group, *n* = 6, respectively) after eight weeks. After surgery, physical status of rats were monitored every day and hemodynamic experiments were also performed in week 11 (Table 1).

Eight weeks after surgery, CHF rats were randomly divided into two groups, each of them with 6 rats; a model group and a SFD treated group. Treated rats were administered SFD (0.5 g of crude botanicals per milliliter of test solution) at a dose of 5 g/kg w.d. orally for 3 weeks from the eighth week. Sham and control rats received the same volume of water each time. After the surgery, 24 h urine was collected at the 11th week after fasting for 12 h. 

### 3.4. Extraction of Metabolites from Urine 

Thirty µL of urine sample were extracted with a mixture of acetonitrile-isopropanol-water (3:3:2) as detailed elsewhere [38]. The extract was then concentrated to dryness in a vacuum evaporator and kept at −80 °C until derivatization. The extract was derivatized prior to analysis with 15 µL of a solution of 40 mg/mL methoxyamine hydrochloride in pyridine at 30 °C for 90 min, and 45 µL of MSTFA was subsequently added at 37 °C for 30 min. A mixture of fatty acid methyl esters was added to the extract as internal retention index markers.

### 3.5. GC–TOF–MS Analysis of Urine Extract

Derivatized samples were analyzed by an Agilent 6890 GC–LECO Pegasus III TOF (LECO, St. Joseph, MI, USA) instrument equipped with a cooled injection system (CIS4, Gerstel, Mülheim an der Ruhr, Germany), an automated linear exchange system (ALEX), and a multi-purpose sampler (MPS) from Gerstel. The injector was run with an initial temperature of 50 °C and ramped to 275 °C at a rate of 12 °C/s. The injection volume was 0.5 µL. GC conditions were set with a programmed oven temperature of 50 °C, held there for 1 min, then increased to 330 °C at a rate of 20 °C/min, and held at 330 °C for 15 min with a carrier gas helium flow rate of 1.0 mL/min. The column was an RTX-5 MS, 30 m long × 0.25 mm i.d., 0.25 mm film. The transfer line and ion source temperature were set at 280 °C and 250 °C, respectively. Solvent delay was adjusted to 5.5 min and MS acquisition was optimized to 17 spectra per scan, using a mass range of 50 to 600 *m*/*z*.

### 3.6. GC–TOF–MS Data Analysis

Spectra over the range of 85–500 *m*/*z* were preprocessed by the Chroma TOF software (Version 2.32, LECO, St. Joseph, MI, USA) using automated peak detection and mass spectral deconvolution. These spectra were further processed using the in-house (UC Davis Genome Center, Davis, CA, USA) programmed database BinBase [39]. By using the algorithm underlying BinBase, inconsistent signals and noise peaks were removed and metabolite signals were structurally identified as known metabolites based on comparison with mass spectra and retention indices of customized reference mass spectral libraries that were acquired using authentic standard compounds under identical data acquisition parameters. The metabolite data were then normalized based on the cell dry weight in each sample.

### 3.7. Statistical Analysis

The resulting data sets were imported into the Statistica software (Version 7.1, StatSoft, Tulsa, OK, USA, 2005) for multivariate statistical analysis and univariate analysis. The unit variance was applied to all multivariate analyses. Principal components analysis (PCA), an unsupervised pattern recognition method, was conducted to examine the intrinsic variation in the data set via Metaboanalyst [40]. Independent *t*-test, hierarchical clustered heat map (MultiExperiment Viewer, 4.8.1, Dana-Farber Cancer Institute, Boston, MA, USA), and analysis of variance (ANOVA) were used to determine if there were significant differences in the marker metabolite levels between classes. Metabolomic pathway analysis combining the modules of pathway enrichment analysis with pathway topology analysis was performed by Metaboanalyst to identify metabolic pathways that are significantly altered in different groups. Rattus norvegicus pathway library was used in the following pathway analysis. The most relevant metabolic pathways in this metabolomics study were identified using a pathway enrichment algorithm called “Global test”, and the impact of each pathway identified was calculated using “Relative Betweeness Centrality”. ANOVA was performed by Statistica. F-statistics and *p*-values were generated for all metabolites. Data distributions are displayed by box-whisker plots, indicating the arithmetic mean value for each category, the standard error as a box, and whiskers for 1.96 times the category standard error to indicate the 95% confidence intervals, assuming normal distributions. 

## 4. Conclusions

In this study, a GC/TOF–MS-based metabolomic study was conducted to increase the understanding of CHF and assess the efficacy and mechanism(s) of SFD in treating CHF induced by coronary artery ligation in rats. The CHF-related urine metabolite profiles of rats with CHF induced by coronary artery ligation were significantly altered in our studies. Aside from any other physiological functions, the molecules differentially excreted between CHF and sham surgery, control and SFD-treated groups are mainly involved in energy metabolism. Treatment with SFD greatly improved the cardiovascular function of CHF rats. This therapeutic effect of SFD could be attributed to restoring the disturbed energy metabolism pathways. These findings can help us increase the understanding of CHF and investigate therapeutic effects and mechanisms of Shenfu decoction. 

## Supplementary Materials

Supplementary materials can be accessed at: http://www.mdpi.com/1420-3049/20/07/11915/s1.
